# Vaginal Microbiome Profiles and Reproductive Outcomes: Implications for Natural Conception and Assisted Reproduction

**DOI:** 10.7759/cureus.115276

**Published:** 2026-08-27

**Authors:** Varsha Prakash, Sarahh Galiotte, Urja Mhatre, Angel Chirayil John, Husnia Ahmadi, Yara Moustafa Manah, Deema Ahmed Ali Elgasim, Aishwarya Sreekumar Nair, Manju Rai

**Affiliations:** 1 Medicine, Sri Ramachandra Institute of Higher Education and Research, Chennai, IND; 2 Obstetrics and Gynaecology, Ross University School of Medicine, New York, USA; 3 Obstetrics and Gynaecology, University of Lancashire, Burnley, GBR; 4 Obstetrics and Gynaecology, Tbilisi State Medical University, Tbilisi, GEO; 5 Obstetrics and Gynaecology, University of California San Diego Medical Center, San Diego, USA; 6 Obstetrics and Gynaecology, Mansoura University, Mansoura, EGY; 7 Obstetrics and Gynaecology, Ain Shams University Hospital, Cairo, EGY; 8 Obstetrics and Gynaecology, ACS Medical College and Hospital, Chennai, IND; 9 Biotechnology, Shri Venkateshwara University, Gajraula, IND

**Keywords:** assisted reproductive technology, dysbiosis, endometrial receptivity, implantation failure, in vitro fertilization, lactobacillus dominance, multi-omics, precision reproductive medicine, reproductive immunology, vaginal microbiome

## Abstract

The vaginal microbiome is increasingly recognized as an important determinant of female reproductive health, with emerging evidence linking its composition and function to fertility, implantation, and pregnancy outcomes. This narrative review synthesizes current evidence on the role of the vaginal microbiome in natural conception and assisted reproductive technology (ART), with emphasis on underlying mechanisms, clinical implications, and emerging therapeutic applications. Contemporary literature examining vaginal microbiome composition, community state types (CSTs), host-microbiome interactions, and reproductive outcomes was qualitatively synthesized alongside evidence from mechanistic studies and emerging multi-omics and artificial intelligence (AI)-based approaches. *Lactobacillus*-dominant profiles, particularly those enriched in *Lactobacillus crispatus*, are often associated with more favorable reproductive outcomes, whereas *Lactobacillus iners*-dominant communities may show more variable associations; dysbiotic, highly diverse microbial profiles have been associated with adverse outcomes including infertility, implantation failure, and pregnancy loss, although these relationships vary according to the *Lactobacillus *species involved, sampling compartment, population studied, and reproductive endpoint. The vaginal microbiome may influence reproductive success through effects on epithelial barrier integrity, immune regulation, inflammatory signaling, microbial metabolite production, sperm function, and endometrial receptivity. Emerging evidence also supports functional interplay between the vaginal and endometrial microbial environments. In natural conception, microbial composition may affect sperm viability, fertilization, and early pregnancy maintenance, whereas in ART, it has been associated with implantation, clinical pregnancy, and live birth outcomes. Multi-omics and AI-based approaches may improve risk stratification and predictive modeling, while microbiome-directed interventions, including probiotics and vaginal microbiota transplantation, remain promising but investigational. The vaginal microbiome therefore represents a potential biomarker and investigational therapeutic target in reproductive medicine; however, routine clinical application remains limited by methodological heterogeneity, inconsistent definitions of dysbiosis, and insufficient interventional evidence. Standardized sampling and analytical methods, together with well-designed prospective and interventional studies, are needed before microbiome profiling and targeted modulation can be integrated into routine fertility care.

## Introduction and background

The vaginal microbiome has undergone a profound conceptual transformation over the past two decades, evolving from a simplistic infection-centered framework to a complex ecological and systems biology paradigm. Historically, culture-based methods supported the notion that a "healthy" vagina was uniformly dominated by *Lactobacillus *species, which were believed to maintain vaginal homeostasis through lactic acid production and pathogen inhibition. However, the advent of high-throughput sequencing technologies, particularly 16S rRNA gene sequencing, has revolutionized this understanding by revealing substantial interindividual variability and identifying distinct microbial configurations termed community state types (CSTs) [1,2]. These CSTs range from *Lactobacillus*-dominant profiles (e.g., *Lactobacillus crispatus* and *Lactobacillus iners*) to more diverse, anaerobe-rich communities, with dynamic transitions influenced by hormonal fluctuations, host immunity, sexual behavior, and genetic factors [1-3]. CSTs therefore provide a framework for describing distinct patterns of vaginal microbial community composition rather than discrete disease categories. Although *L. crispatus*-dominant CSTs are generally characterized by greater lactic acid production and a low-pH environment, other *Lactobacillus *species, particularly *L. iners*, have distinct metabolic and ecological characteristics and show less consistent associations with reproductive outcomes. Importantly, most evidence linking CST composition with fertility and assisted reproductive technology (ART) outcomes remains observational, and causal relationships have not yet been established.

This paradigm shift has exposed critical limitations in traditional infection-based models that primarily focus on overt clinical entities such as bacterial vaginosis (BV), vulvovaginal candidiasis, and aerobic vaginosis. While these conditions remain clinically relevant, subtle asymptomatic shifts in vaginal microbial composition have sometimes been described as "subclinical dysbiosis"; however, this term lacks a universally accepted diagnostic threshold and should be regarded as a descriptive research concept rather than a defined clinical diagnosis [3,4]. The conventional binary classification of "healthy" versus "infected" vaginal states fails to capture this complexity and overlooks the nuanced interactions between microbial communities and host reproductive function. Consequently, there is increasing recognition that vaginal microbiota play a broader role beyond infection, influencing mucosal immunity, epithelial integrity, and local inflammatory responses that are critical for successful conception and implantation [4,5].

The clinical relevance of this evolving understanding is underscored by the rising global burden of infertility and the persistent limitations of ART. Despite significant technological advancements in in vitro fertilization (IVF) and intracytoplasmic sperm injection (ICSI), implantation failure and unexplained infertility remain major challenges, affecting a substantial proportion of couples undergoing treatment. These gaps in clinical success suggest the involvement of previously underappreciated biological factors, among which the reproductive tract microbiome has emerged as a key candidate [5,6]. Notably, infertility and unfavorable reproductive outcomes have been associated with vaginal dysbiosis characterized by reduced *Lactobacillus *dominance and increased abundance of anaerobic bacteria, whereas *Lactobacillus*-dominant microbiota are generally linked with improved implantation rates, pregnancy success, and live birth outcomes [6,7].

In parallel, growing evidence highlights the interconnectedness of the vaginal and upper reproductive tract microbiomes, often referred to as the "vaginal-uterine axis". Alterations in vaginal microbial composition may influence endometrial receptivity, embryo implantation, and early pregnancy maintenance through ascending microbial migration, immune modulation, and metabolic signaling pathways [7,8]. Prospective evidence from the ReceptIVFity study suggests that vaginal microbiome profiles may help stratify pregnancy outcomes following ART, supporting further investigation of microbiome profiles as potential reproductive biomarkers [9].

Recent advances in multi-omics technologies, including metagenomics, metabolomics, transcriptomics, and proteomics, are providing deeper insights into the functional and mechanistic roles of the vaginal microbiome. These approaches, when integrated with artificial intelligence (AI)-driven analytical models, enable the high-resolution characterization of microbial-host interactions and facilitate the identification of predictive signatures associated with reproductive success or failure [3,4]. Such innovations are paving the way for precision reproductive medicine, where individualized microbiome profiling may guide diagnostic stratification and therapeutic interventions, including targeted probiotics, microbiome modulation, and personalized ART protocols.

In this context, the present review aims to comprehensively synthesize current evidence on vaginal microbiome profiles and their implications for natural conception and assisted reproduction. We explore the ecological dynamics of the vaginal microbiome, its interaction with host immune and endocrine pathways, and its role in shaping reproductive outcomes. Furthermore, we examine emerging therapeutic strategies and technological advancements, including multi-omics and AI-based approaches, while critically addressing existing controversies and translational challenges. By integrating these perspectives, this review seeks to advance the understanding of microbiome-driven reproductive health and highlight its potential in shaping the future of personalized fertility care.

## Review

Methodology

This narrative review was conducted to provide a comprehensive and up-to-date synthesis of current evidence on the role of the vaginal microbiome in natural conception and assisted reproductive outcomes. A structured literature search was performed across major electronic databases, including PubMed/MEDLINE, Scopus, Web of Science, and the Cochrane Library, to identify relevant studies published between January 2015 and March 5, 2026. Additional seminal and foundational studies published prior to this period were included where necessary to provide historical context.

The search strategy incorporated a combination of Medical Subject Headings (MeSH) terms and free-text keywords related to the vaginal microbiome and reproductive outcomes. Key search concepts included "vaginal microbiome," "vaginal microbiota", "community state types", "dysbiosis", "Lactobacillus", "fertility", "natural conception", "in vitro fertilization", "intracytoplasmic sperm injection", "endometrial microbiome", "implantation failure", and "reproductive outcomes". Boolean operators ("AND", "OR") were used to combine these concepts. The final database searches were conducted on March 5, 2026. Database-specific search strategies were adapted according to the indexing and search syntax of each database. Database-specific search strategies based on the documented search concepts are provided in the Appendices for transparency and reproducibility. All retrieved records were imported into Zotero Version 10 (Corporation for Digital Scholarship, Vienna, Virginia, United States) for reference management and duplicate removal. Duplicate records were identified and removed using Zotero's duplicate-detection function before title and abstract screening.

Studies were considered eligible if they evaluated the composition, function, or clinical implications of the vaginal or endometrial microbiome in relation to fertility, pregnancy outcomes, or ART success. Both observational and interventional studies, including cohort studies, case-control studies, randomized controlled trials, and systematic reviews, were included. Experimental and translational studies exploring mechanistic insights, including multi-omics analyses, were also considered. Articles not published in English, conference abstracts without full text, and studies lacking clear methodological descriptions were excluded.

During qualitative synthesis, evidence was interpreted according to study design and evidentiary role, with prospective cohort studies, randomized controlled trials, and other clinical outcome studies given greater weight for conclusions regarding reproductive outcomes and clinical implications, while observational, mechanistic, and translational studies were primarily used to contextualize biological plausibility and generate hypotheses. Systematic reviews were considered alongside the underlying evidence and used to support broader patterns rather than to override higher-quality primary clinical evidence.

Two reviewers independently screened titles and abstracts for relevance, followed by full-text evaluation of selected articles. Discrepancies were resolved through discussion and consensus. Given the narrative nature of this review, formal quantitative synthesis or meta-analysis was not performed; instead, findings were synthesized qualitatively, with emphasis on identifying consistent patterns, mechanistic insights, and areas of controversy.

To ensure scientific rigor, priority was given to recent studies, particularly those published within the last 5-10 years, as well as landmark trials and widely cited foundational research. Given the narrative nature of this review, no formal risk-of-bias or methodological quality assessment was performed. Additionally, emerging evidence from multi-omics studies and AI-based predictive models was included to highlight evolving trends in precision reproductive medicine. The synthesis was structured thematically to align with key domains, including microbiome architecture, host-immune interactions, natural conception, ART outcomes, therapeutic modulation, and translational challenges.

Study Selection Process (Preferred Reporting Items for Systematic Reviews and Meta-Analyses (PRISMA)-Based Narrative Framework)

**Figure 1:**
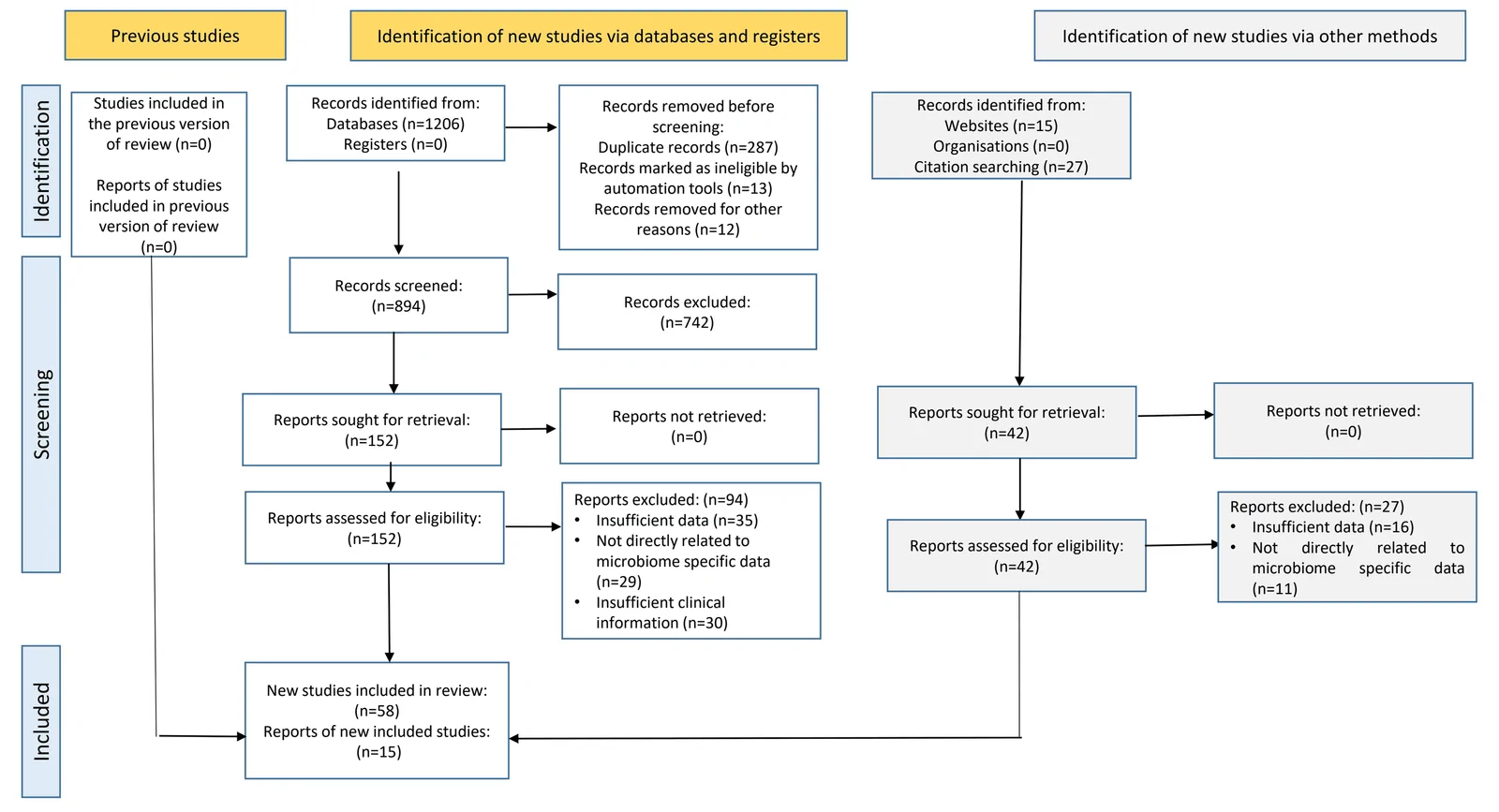
Flow diagram of the study selection process

The study selection process followed PRISMA-based principles adapted for a narrative review. A total of 1,206 records were identified through database searches, while an additional 42 records were identified through website and citation searching (Figure 1). After the removal of 287 duplicate records, 13 records marked as ineligible by automation tools, and 12 records removed for other reasons, 894 records underwent title and abstract screening. Of these, 742 records were excluded due to irrelevance or failure to meet the eligibility criteria. The remaining 152 full-text articles were assessed for eligibility, and 94 reports were excluded because of insufficient data (n=35), lack of microbiome-specific information (n=29), or insufficient clinical information (n=30). In parallel, 42 reports identified through citation searching were retrieved and assessed for eligibility, of which 27 were excluded owing to insufficient data (n=16) and lack of direct relevance to microbiome-specific data (n=11). Ultimately, 58 studies identified through database searches, together with 15 studies identified through website and citation searching, were included in the qualitative synthesis, yielding a total of 73 included studies comprising observational studies, randomized controlled trials, and translational research.

Vaginal microbiome architecture: CSTs and beyond

The structural and functional organization of the vaginal microbiome has been most commonly described using the framework of CSTs, originally proposed through high-resolution sequencing studies. These CSTs classify vaginal microbial communities into five major categories: four *Lactobacillus*-dominant groups (*L. crispatus*, *L. gasseri*, *L. iners*, and *L. jensenii*) and one diverse, non-*Lactobacillus*-dominant group enriched with anaerobic bacteria such as *Gardnerella*, *Atopobium*, *Prevotella*, and *Mobiluncus *[10,11]. Among these, *L. crispatus*-dominated CSTs are commonly associated with a lower-risk vaginal microbial state, potentially reflecting robust lactic acid production, maintenance of low vaginal pH, and inhibition of pathogenic colonization. In contrast, non-*Lactobacillus*-dominant CSTs are often associated with greater microbial diversity and, in some populations, increased inflammatory activity and adverse reproductive outcomes; however, such communities may also occur asymptomatically, and their clinical significance varies across populations [11,12].

However, the CST framework, while foundational, represents a primarily taxonomic classification that does not fully capture the functional complexity of microbial ecosystems. Increasing evidence suggests that microbial function, rather than mere composition, plays a more critical role in determining reproductive health outcomes. For instance, different *Lactobacillus *species exhibit distinct metabolic and immunomodulatory properties; *L. crispatus *is associated with sustained lactic acid production and bacteriocin activity, whereas *L. iners*, despite being classified as *Lactobacillus*-dominant, may coexist with dysbiotic communities and produce fewer protective metabolites [12,13]. This functional heterogeneity challenges the simplistic dichotomy of "beneficial" versus "harmful" taxa and underscores the need to shift toward a function-centric understanding of the vaginal microbiome.

At a deeper level, strain-specific variation further complicates this landscape. Genomic analyses have revealed substantial intra-species diversity, with different strains of the same species exhibiting divergent metabolic capacities, virulence potential, and interactions with host immunity [13,14]. For example, certain *Gardnerella vaginalis *strains possess enhanced biofilm-forming capabilities and cytotoxic properties, while others may exist as commensals with limited pathogenicity [14]. Similarly, strain-level differences in *Lactobacillus *species influence their ability to produce D- and L-lactic acid isomers, which have differential effects on epithelial barrier function and immune signaling [13]. These findings highlight the importance of moving beyond species-level classification toward high-resolution genomic and functional profiling.

Biofilm formation represents another critical dimension of vaginal microbiome architecture. Dysbiotic states, particularly those associated with BV, are characterized by the formation of polymicrobial biofilms dominated by *Gardnerella *and other anaerobes [15]. These biofilms confer resistance to host immune responses and antimicrobial therapies, contributing to recurrent infections and persistent microbial instability. Importantly, biofilm-associated bacteria, particularly in BV, can form adherent communities on the vaginal epithelium and may contribute to persistence of dysbiotic states [15,16]. Such microbial alterations may influence local epithelial and inflammatory environments, providing a plausible pathway through which vaginal dysbiosis could affect reproductive processes; however, direct effects of vaginal biofilms on sperm function, embryo implantation, or early pregnancy maintenance remain insufficiently established. The resilience of these biofilms further complicates therapeutic interventions and underscores the need for targeted anti-biofilm strategies.

Temporal dynamics add another layer of complexity to vaginal microbiome architecture. Contrary to earlier assumptions of relative stability, longitudinal studies have demonstrated that the vaginal microbiome is highly dynamic, with fluctuations occurring across the menstrual cycle, sexual activity, hormonal changes, and environmental exposures [16,17]. Estrogen-driven glycogen deposition in vaginal epithelial cells promotes *Lactobacillus *proliferation, particularly during the follicular and luteal phases, whereas menstruation is often associated with transient increases in microbial diversity and reduced *Lactobacillus *dominance [17]. These cyclical variations may have important implications for the timing of conception and assisted reproductive interventions, suggesting that microbiome assessment should be contextualized within temporal and hormonal frameworks.

Studies have reported differences in the distribution of vaginal CSTs across populations with different reported racial or ethnic backgrounds, including a greater prevalence of non-*Lactobacillus*-dominant CSTs in some populations of African and Hispanic ancestry. These patterns are likely shaped by complex interactions among biological, behavioral, socioeconomic, environmental, cultural, and healthcare-related factors and should not be interpreted as evidence of intrinsic race-based biological differences [11,18]. Importantly, such variability challenges the universal applicability of a single "optimal" microbiome profile and highlights the need for population-specific reference standards in both research and clinical practice [18].

Despite the transformative insights provided by 16S rRNA gene sequencing, this approach has inherent limitations that restrict its clinical utility. While it enables broad taxonomic profiling, it lacks the resolution to differentiate closely related species and strains, provides limited functional information, and is susceptible to biases related to primer selection and sequencing depth [19]. Furthermore, 16S-based methods cannot reliably detect non-bacterial components of the microbiome, including viruses, fungi, and archaea, which may also play important roles in reproductive health [19]. These limitations have prompted a shift toward more comprehensive and integrative methodologies.

Shotgun metagenomic sequencing has emerged as a powerful alternative, offering high-resolution insights into microbial composition, gene content, and functional potential. Recent studies (2020-2025) have leveraged metagenomics to identify metabolic pathways associated with vaginal health and disease, including those involved in lactic acid production, amino acid metabolism, and immune modulation [13,20]. Moreover, integration with metabolomic and transcriptomic data has enabled the characterization of host-microbe interactions at unprecedented depth, revealing functional signatures associated with implantation success, pregnancy outcomes, and ART responsiveness [20,21]. These multi-omics approaches are facilitating a paradigm shift from descriptive microbiology to mechanistic and predictive modeling.

In this evolving landscape, the focus of vaginal microbiome research is shifting from descriptive taxonomic classification toward functional characterization. While community composition provides important ecological context, emerging evidence indicates that microbial activity, metabolite production, and host-microbe interactions are more directly relevant to reproductive outcomes. This functional perspective is particularly important in reproductive medicine, where microbial signaling pathways may influence endometrial receptivity and early pregnancy events. As discussed in later sections, integrative multi-omic approaches are increasingly being used to capture these dynamics and to support clinically relevant predictive modeling.

Host-microbiome-immune crosstalk in the reproductive tract

The interplay between the vaginal microbiome and host immune system represents a potential contributor to reproductive success, functioning as a dynamic interface that regulates mucosal homeostasis, implantation tolerance, and early pregnancy maintenance. Rather than acting as passive colonizers, microbial communities actively shape immune responses within the female reproductive tract, influencing epithelial integrity, cytokine signaling, and adaptive immune regulation. Increasing evidence suggests that immune modulation serves as a central mediator linking microbiome composition to fertility outcomes, particularly in the context of implantation failure and recurrent pregnancy loss [22].

A key component of this interaction is the integrity of the vaginal epithelial barrier, which serves as the first line of defense against pathogenic invasion. *Lactobacillus*-dominant microbiota contribute to barrier stability through the production of lactic acid, maintenance of acidic pH, and enhancement of tight junction protein expression. In contrast, dysbiosis characterized by anaerobic overgrowth disrupts epithelial integrity, increases permeability, and facilitates microbial translocation into the upper reproductive tract [22,23]. This disruption not only predisposes to infection but also triggers local immune activation, thereby creating a pro-inflammatory microenvironment that may impair sperm viability, embryo implantation, and endometrial receptivity.

Cytokine and chemokine signaling pathways represent another critical axis of host-microbiome interaction. Eubiotic microbial communities are associated with balanced production of anti-inflammatory cytokines such as interleukin (IL)-10 and transforming growth factor-beta (TGF-β), which promote immune tolerance and tissue homeostasis. Conversely, vaginal dysbiosis is linked to elevated levels of pro-inflammatory mediators including IL-1β, IL-6, tumor necrosis factor-alpha (TNF-α), and chemokines that recruit immune cells to the site of inflammation [22,24]. This shift toward a pro-inflammatory cytokine milieu has been implicated in impaired endometrial receptivity and increased risk of implantation failure, particularly in women undergoing ART [24].

Central to successful implantation is the establishment of immune tolerance toward the semi-allogeneic embryo, a process heavily mediated by regulatory T (Treg) cells. The microbiome plays a pivotal role in modulating Treg cell differentiation and function through microbial metabolites and antigenic stimulation. Perturbations in the microbiome-Treg axis can disrupt immune tolerance, leading to an imbalance favoring effector immune responses and increased risk of implantation failure [25]. Recent human observational studies have reported associations between dysbiotic vaginal or endometrial microbiota, altered inflammatory cytokine profiles, and immune dysregulation involving uNK and Treg populations in women with recurrent implantation failure. These findings provide biologically plausible mechanistic links between microbiome alterations and impaired reproductive immune homeostasis, although causal relationships remain unestablished [25].

The balance between T-helper 1 (Th1) and T-helper 2 (Th2) immune responses further illustrates the complexity of microbiome-mediated immune regulation. A successful pregnancy is generally associated with a Th2-dominant, anti-inflammatory environment that supports fetal tolerance. However, microbial dysbiosis can skew this balance toward a Th1-dominant, pro-inflammatory state characterized by the increased production of interferon-gamma (IFN-γ) and other inflammatory mediators [24,26]. Such immune dysregulation has been associated with adverse reproductive outcomes, including implantation failure and recurrent miscarriage. Rather than being governed by a simple Th1/Th2 balance, reproductive immune homeostasis reflects dynamic and temporally regulated interactions among multiple innate and adaptive immune populations. Tregs contribute to immune tolerance, while uterine NK cells, macrophages, and dendritic cells support trophoblast invasion, vascular remodeling, tissue homeostasis, and local immune regulation. Th17 cells and other decidual immune populations also have context-dependent roles in antimicrobial defense, tissue remodeling, and inflammatory regulation. Thus, microbiome-immune interactions may influence reproductive physiology through the modulation of this complex cellular network, although the extent and direction of these effects may vary across reproductive stages and remain incompletely defined [27,28].

Innate immune defenses, particularly antimicrobial peptides (AMPs), also play a crucial role in host-microbiome interactions. Vaginal epithelial cells produce AMPs such as defensins, cathelicidins, and secretory leukocyte protease inhibitors, which provide broad-spectrum antimicrobial activity while maintaining microbial balance. *Lactobacillus *species can enhance AMP production, thereby reinforcing mucosal defense mechanisms. In contrast, dysbiotic microbial communities may downregulate AMP expression or evade their activity through biofilm formation, contributing to persistent inflammation and microbial instability [23,26].

Mucosal immunoglobulin A (IgA) represents another important component of immune regulation within the reproductive tract. Secretory IgA facilitates immune exclusion by binding to microbial antigens and preventing their adherence to epithelial surfaces, thereby maintaining microbial homeostasis. Emerging evidence suggests that the vaginal microbiome influences IgA production and specificity, with eubiotic communities promoting effective immune surveillance and dysbiotic states associated with impaired IgA-mediated protection [22,29]. This interaction further highlights the bidirectional relationship between microbial composition and host immunity.

The cumulative effect of these immune interactions is reflected in the inflammatory microenvironment of the endometrium, which plays a vital role in implantation and early pregnancy. A finely regulated balance between pro- and anti-inflammatory signals is essential for endometrial receptivity, facilitating embryo apposition, adhesion, and invasion. However, microbial dysbiosis can disrupt this balance, leading to chronic low-grade inflammation that impairs endometrial function [23,30]. Studies have demonstrated that increased microbial diversity and loss of *Lactobacillus *dominance are associated with elevated pro-inflammatory cytokines, reduced epithelial integrity, and compromised implantation success [22,30].

One clinically relevant condition in the context of microbiome-immune interactions is chronic endometritis (CE), a clinicopathological condition characterized by persistent endometrial inflammation, typically assessed by histopathological or immunohistochemical evidence of endometrial stromal plasma cells. Although CE may be associated with alterations in the endometrial microbiome, microbiome-defined dysbiosis should not be considered synonymous with CE [23]. CE has been increasingly associated with infertility, recurrent implantation failure, and recurrent pregnancy loss. Alterations in the endometrial microbiome have been proposed as a potential contributor to CE through the modulation of local immune responses, including changes in plasma cell and lymphocyte populations and inflammatory signaling [23,31]. Studies integrating microbiome profiling with transcriptomic analysis have identified distinct microbial and immune signatures associated with CE, supporting a potential relationship between microbial alterations, endometrial inflammation, and impaired reproductive outcomes [31].

Collectively, these findings underscore the central role of immune modulation as the mechanistic bridge between the vaginal microbiome and reproductive success. The intricate crosstalk between microbial communities and host immune pathways not only maintains mucosal homeostasis but may influence the delicate balance between tolerance and inflammation required for successful implantation and pregnancy maintenance. Future research integrating multi-omics approaches and immune profiling is essential to unravel these complex interactions and to develop targeted therapeutic strategies aimed at restoring immune equilibrium and optimizing fertility outcomes.

**Figure 2:**
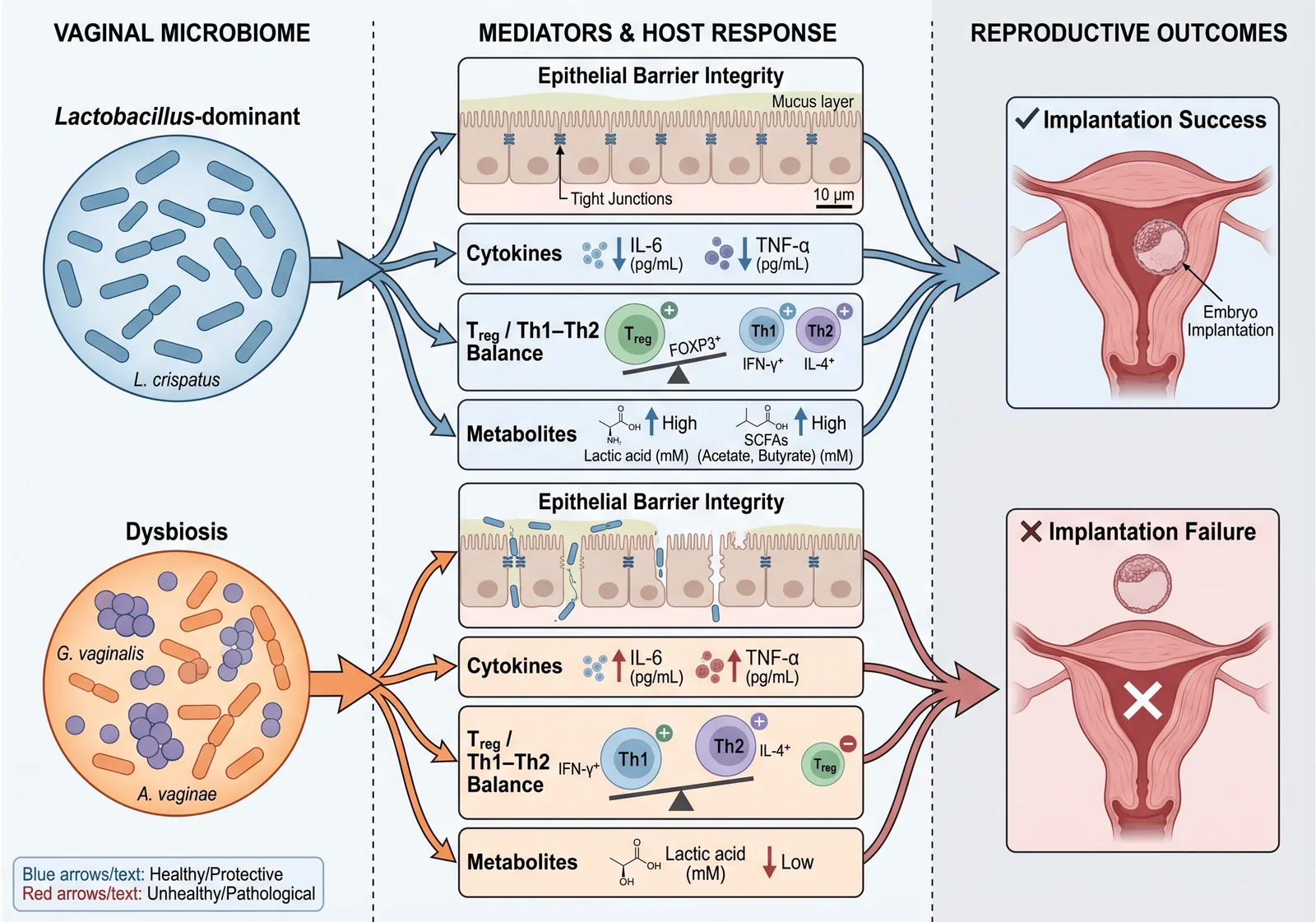
Proposed mechanistic pathways linking vaginal microbiome composition to reproductive outcomes Schematic representation of potential pathways through which *Lactobacillus*-dominant and dysbiotic vaginal microbial communities may influence reproductive physiology. *Lactobacillus*-dominant communities are associated with low vaginal pH through lactic acid production and may contribute to epithelial barrier integrity and local immune regulation. In contrast, dysbiotic communities characterized by the increased abundance of anaerobic bacteria may be associated with altered microbial metabolites, inflammatory signaling, epithelial disruption, and changes in reproductive tract immune responses. These pathways provide a proposed mechanistic framework linking vaginal microbial composition with implantation and pregnancy outcomes; however, the strength of evidence varies across individual pathways, and several downstream relationships are based on mechanistic inference or observational associations rather than established causal effects in humans. The figure has been created by Sarahh Galiotte manually using the standard graphical tools available within BioRender (BioRender Inc., Toronto, Ontario, Canada).

The mechanistic pathways linking vaginal microbiome composition to reproductive outcomes, including immune modulation, epithelial integrity, and inflammatory signaling, are illustrated in Figure 2.

Vaginal microbiome and natural conception

The vaginal microbiome may contribute to natural conception by modulating the physicochemical and immunological environment required for sperm survival, fertilization, and early embryonic development. While traditionally considered primarily in the context of infection, emerging evidence highlights its direct functional influence on gamete interaction and early reproductive success, extending beyond overt dysbiosis to include subtle microbial and metabolic perturbations.

One of the most immediate effects of the vaginal microbiome on fertility is its impact on sperm motility and survival. A *Lactobacillus*-dominant environment, characterized by acidic pH and production of lactic acid and hydrogen peroxide, provides protection against pathogenic organisms but may exert a dual effect on sperm function. While physiological acidity is essential for maintaining vaginal homeostasis, excessive acidification or microbial adhesion to spermatozoa may reduce motility and impair sperm progression through cervical mucus [32,33]. In contrast, dysbiotic states associated with BV are characterized by elevated pH and increased abundance of anaerobic bacteria, which produce cytotoxic metabolites and inflammatory mediators that directly compromise sperm viability and function [32]. Experimental studies have demonstrated that certain vaginal microorganisms can adhere to sperm surfaces, alter membrane integrity, and disrupt intracellular calcium signaling, thereby impairing motility and capacitation [33].

Beyond direct microbial interactions, microbial metabolites play a crucial role in shaping the fertilization environment. Lactic acid produced by *Lactobacillus *species maintains an acidic milieu that limits pathogen colonization while supporting sperm selection mechanisms. Dysbiotic vaginal microbial communities, particularly those associated with BV, have been linked to altered microbial metabolite profiles, including increased production of short-chain fatty acids (SCFAs) such as acetate, propionate, and butyrate, which may influence the local inflammatory environment [34]. Importantly, emerging human evidence suggests that alterations in the vaginal microbiome may also be associated with partner sperm function; a retrospective study of 64 couples found significantly lower progressive sperm motility among couples with an altered vaginal microbiome [35]. However, the specific mechanisms linking vaginal microbial metabolites to sperm dysfunction, including potential effects on mitochondrial function and oxidative stress, remain insufficiently established. Additionally, bacterial products such as lipopolysaccharides (LPS) and cytotoxins (e.g., vaginolysin) have been shown to impair sperm capacitation, acrosomal reaction, and fertilization potential [36]. Sialidase-producing bacteria, commonly enriched in BV, further disrupt sperm function by degrading the glycocalyx, thereby increasing susceptibility to immune-mediated damage and reducing sperm transit through cervical mucus [36]. Collectively, these findings underscore the importance of microbial metabolic activity as a determinant of fertilization success.

Vaginal dysbiosis has also been increasingly linked to early reproductive failure, particularly biochemical pregnancy loss and early pregnancy loss. Clinical studies have demonstrated that women with reduced *Lactobacillus *dominance and increased microbial diversity exhibit higher rates of early pregnancy loss, even in the absence of overt clinical symptoms [37]. Specific taxa such as *Streptococcus *and *Porphyromonas *have been associated with early pregnancy loss, whereas *L. crispatus* has been associated with a lower risk of adverse pregnancy outcomes in some studies, although these associations remain observational and may vary across populations and study settings [37]. These associations are thought to be mediated through microbial-induced inflammation, cytokine dysregulation, and impaired endometrial receptivity, linking vaginal microbiome composition to early embryonic survival.

Recurrent miscarriage represents another clinically significant manifestation of microbiome-related reproductive dysfunction. Women with recurrent miscarriage have been shown to exhibit increased vaginal microbial diversity, higher prevalence of pathogenic organisms, and reduced stability of microbial communities [38]. Mechanistically, dysbiosis-driven activation of chemokine pathways and local immune responses can disrupt uterine microcirculation and impair implantation processes [38]. Furthermore, microbial-induced inflammation may alter the delicate immune tolerance required for early pregnancy maintenance, thereby contributing to recurrent pregnancy loss. These findings suggest that alterations in vaginal microbiome composition may represent a potential contributor or biomarker of reproductive dysfunction rather than an established determinant of pregnancy viability.

Given these associations, preconception optimization of the vaginal microbiome has emerged as a potential strategy to improve natural fertility outcomes. Probiotic interventions, particularly those containing *Lactobacillus *strains such as *Lactobacillus rhamnosus *GR-1 and *Lactobacillus reuteri *RC-14, have demonstrated efficacy in restoring microbial balance and reducing the recurrence of dysbiosis [39]. Emerging approaches such as vaginal microbiota transplantation and targeted microbiome modulation are being explored as novel therapeutic strategies, although robust clinical evidence remains limited. Importantly, the timing and patient selection for such interventions require careful consideration, as excessive or inappropriate modulation may disrupt physiological microbial balance and potentially affect sperm function.

A particularly emerging concept in reproductive microbiology is the potential interplay between partners' genital microbiomes and its possible relevance to natural conception. Preliminary couple-level evidence suggests that vaginal microbiome composition may be associated with partner sperm characteristics [35], although prospective studies directly evaluating microbiome interactions and natural conception are needed. The vaginal and seminal microbiomes are distinct yet interconnected ecosystems, with microbial exchange occurring during sexual intercourse. Studies have demonstrated that semen can transiently alter the vaginal microbiome by increasing pH and introducing exogenous microbial species, particularly in cases of unprotected intercourse [40]. This interaction is bidirectional, with the vaginal microbiome also influencing seminal parameters, including sperm motility and viability.

Recent research has introduced the concept of a "complementary seminovaginal microbiome", suggesting that microbial compatibility between partners may influence fertility outcomes. Dysbiosis in one partner may adversely affect the reproductive environment of the other, thereby reducing the likelihood of successful conception [40,41]. For example, male partners with altered seminal microbiota have been associated with reduced sperm motility in couples where female partners exhibit vaginal dysbiosis, highlighting a synergistic effect of microbial imbalance [40]. Furthermore, seminal microbiota may modulate female immune responses, influencing tolerance mechanisms and potentially affecting fertilization and implantation.

Collectively, these findings highlight that natural conception is influenced not only by individual reproductive physiology but also by the surrounding microbial environment and its interaction with both partners. Rather than acting solely through overt infection, the vaginal microbiome shapes sperm function, fertilization efficiency, and early embryonic viability through a combination of physicochemical conditions and microbial metabolites. This broader, couple-centered perspective highlights the potential value of investigating microbiome assessment in unexplained infertility. The relationship between vaginal microbiome profiles and natural conception outcomes is summarized in Table 1.

**Table 1:** Microbiome profiles and their impact on natural conception and early reproductive outcomes CST: community state type; BV: bacterial vaginosis; SCFAs: short-chain fatty acids; LPS: lipopolysaccharide

Microbiome profile	Key characteristics	Impact on natural conception	Underlying mechanisms	Reference(s)
*Lactobacillus crispatus*-dominant	Low pH, high lactic acid production	↑ sperm survival, ↑ fertilization success, ↓ early pregnancy loss	Maintains acidic environment, reduces pathogen colonization, supports immune tolerance	[32,34]
*Lactobacillus iners*-dominant	Transitional, less stable microbiota	Variable fertility outcomes	Reduced protective metabolite production, coexistence with dysbiosis	[12,13]
Dysbiotic (anaerobe-dominant; BV-like)	High microbial diversity, increased *Gardnerella*, *Prevotella*	↓ sperm motility, ↑ biochemical pregnancy loss, ↑ miscarriage risk	Production of SCFAs, LPS, cytotoxins, increased inflammation	[32,36,37]
Biofilm-associated microbiota	Polymicrobial biofilm formation	Impaired fertilization and sperm penetration	Biofilm resistance, epithelial disruption, inflammatory activation	[15,16]
Unstable/mixed CST	Temporal fluctuations in microbiota	Unpredictable conception outcomes	Hormonal influence, microbial instability, immune variability	[16,17]

Vaginal microbiome in assisted reproduction (IVF/ICSI/FET)

ART, including IVF, ICSI, and frozen embryo transfer (FET), has revolutionized infertility management. However, implantation failure and suboptimal live birth rates remain persistent challenges. Increasing attention has therefore shifted toward previously underrecognized biological contributors, including the reproductive tract microbiome, which appears to influence key stages of ART beyond conventional embryological factors.

Microbiome Profiles and ART Outcomes Across Stages

Oocyte quality and follicular environment: Emerging evidence suggests that the reproductive tract microbiome may influence oocyte competence through its interaction with the follicular microenvironment. Microorganisms have been detected in follicular fluid, and their composition appears to correlate with oocyte quality and subsequent embryological outcomes. In a prospective cohort study, the presence of *Lactobacillus *species within follicular fluid was associated with improved embryo development and higher likelihood of embryo transfer, whereas non-*Lactobacillus *taxa were linked with poorer reproductive outcomes [42]. These findings suggest that microbial translocation or systemic microbial signatures may influence oocyte maturation, although causality remains uncertain.

Fertilization rates: Data linking vaginal microbiome composition directly to fertilization rates remain limited and heterogeneous. Some studies suggest that dysbiosis-associated inflammation and oxidative stress may impair gamete interaction and fertilization efficiency. However, observational studies, including those evaluating recurrent implantation failure cohorts, have reported no significant differences in fertilization rates between women with *Lactobacillus*-dominant and *Lactobacillus*-non-dominant microbiota [43]. This indicates that microbiome effects may be more pronounced at later stages of implantation rather than at fertilization itself.

Embryo quality and grading: The relationship between the vaginal microbiome and embryo morphology or grading remains inconclusive. Available data suggest that early embryological parameters, including embryo number and morphology, are not consistently affected by vaginal microbial composition [43]. However, indirect effects mediated through follicular fluid microbiota or systemic inflammation may influence embryo competence. The lack of consistent associations likely reflects methodological heterogeneity and the multifactorial nature of embryo development.

Implantation success: The strongest and most consistent evidence linking the microbiome to ART outcomes pertains to implantation. As discussed earlier, *Lactobacillus*-dominant reproductive tract profiles are associated with enhanced endometrial receptivity and implantation success. In a landmark prospective study, women with a *Lactobacillus*-dominant (>90%) endometrial microbiota exhibited significantly higher implantation, clinical pregnancy, and live birth rates compared with those with dysbiotic profiles [44]. Similarly, vaginal microbiome studies have demonstrated that reduced *Lactobacillus *abundance is associated with lower implantation rates following IVF [9]. These findings support the concept that microbial composition influences the uterine microenvironment and embryo-endometrium interaction.

Clinical pregnancy and live birth rates: Beyond implantation, vaginal microbiome composition has also been associated with downstream reproductive outcomes, although the direction and clinical significance of these associations remain incompletely established. Several studies have reported that *Lactobacillus*-dominant vaginal or endometrial microbiota are associated with higher clinical pregnancy and live birth rates in ART cycles [9,44]. Conversely, dysbiosis characterized by increased microbial diversity and anaerobic overgrowth is associated with reduced ongoing pregnancy rates and increased risk of early pregnancy loss. These associations highlight the potential of microbiome profiling as a prognostic tool in ART.

Dysbiosis and Recurrent Implantation Failure

Recurrent implantation failure represents a major clinical challenge in ART, and increasing evidence implicates microbial dysbiosis as a contributing factor. Women with recurrent implantation failure exhibit altered microbial diversity, reduced *Lactobacillus *dominance, and shifts in key bacterial taxa within both vaginal and endometrial compartments [45]. Importantly, dysbiosis in recurrent implantation failure is not limited to simple depletion of *Lactobacillus *but involves broader ecological disruption and functional imbalance.

Studies analyzing paired vaginal and endometrial samples have demonstrated that microbial alterations may be compartment-specific, suggesting differential roles of local microbiota in implantation biology [46]. Moreover, dysbiosis-associated inflammation and immune dysregulation, discussed in earlier sections, may impair endometrial receptivity and contribute to implantation failure. Despite these associations, the causal relationship remains incompletely understood, and current evidence is largely observational.

Timing of Microbiome Sampling in ART Cycles

The timing of microbiome assessment represents a critical methodological and clinical consideration. Studies have sampled the microbiome at different stages of ART, including before treatment and in close temporal proximity to embryo transfer, contributing to variability in reported findings [43,44,46].

Sampling before ovarian stimulation may provide a baseline assessment of microbial status and facilitate the investigation of whether microbiome characteristics could inform future pre-treatment strategies [44]. However, hormonal changes occurring during ART treatment may alter the reproductive tract environment and microbial composition, potentially limiting the predictive value of a single early assessment. Conversely, sampling in close proximity to embryo transfer may better reflect the microbial environment relevant to implantation, but provides limited opportunity for therapeutic intervention before transfer [43,46].

This temporal variability underscores the need for standardized sampling protocols and longitudinal studies to determine the most clinically relevant time points for microbiome assessment [43,44,46].

Predictive Value of CST Classification in ART

As discussed previously, vaginal microbiome profiles can be categorized into CSTs, with *Lactobacillus-*dominant CSTs generally associated with favorable reproductive outcomes. In the context of ART, CST classification has shown potential as a predictive biomarker for treatment success.

Prospective studies have demonstrated that non-*Lactobacillus*-dominant CSTs are associated with significantly lower implantation and pregnancy rates following IVF [44]. Predictive models incorporating CST classification have shown promise in identifying patients at increased risk of ART failure. However, variability in CST definitions, sequencing methodologies, and population characteristics currently limits their routine clinical application.

Integration of Microbiome Testing into ART Decision Points

A key emerging paradigm in reproductive medicine is the integration of microbiome assessment into ART clinical workflows, enabling a transition toward personalized and precision-based care. Within this framework, microbiome profiling has the potential to inform decision-making at multiple stages of the ART cycle. In the pre-stimulation phase, baseline evaluation of the vaginal or endometrial microbiome may facilitate the identification of dysbiosis prior to ovarian stimulation, thereby allowing for targeted interventions such as probiotics, selective antimicrobial therapy, or lifestyle modifications. Such pre-treatment optimization may help establish a more favorable reproductive tract environment before the initiation of ART [44,47].

As the cycle progresses, microbiome assessment immediately prior to embryo transfer may provide additional information about the endometrial microbial environment and its potential association with reproductive outcomes. In cases where significant dysbiosis is detected, postponement of embryo transfer, particularly in elective or freeze-all strategies, may be considered to allow for microbiome modulation. This approach is especially relevant in frozen embryo transfer cycles, where the flexibility in timing offers a unique opportunity to correct microbial imbalance before implantation. Restoration of a *Lactobacillus*-dominant environment during this window may enhance implantation potential and improve overall ART outcomes [43,44].

Beyond individual time points, advances in AI and multi-omics technologies are further expanding the potential clinical utility of microbiome data. Integration of microbial profiles with clinical, hormonal, and embryological parameters may enable the development of predictive models capable of stratifying patients based on implantation probability and likelihood of treatment success. Such models could ultimately guide individualized treatment strategies, including selection of optimal timing for embryo transfer and targeted microbiome interventions [3,20].

Despite these promising developments, microbiome-guided ART remains largely investigational. Significant challenges persist, including the lack of standardized diagnostic thresholds for defining dysbiosis, variability in sequencing methodologies, and limited interventional data demonstrating clear improvements in clinical outcomes. Consequently, while microbiome profiling represents a compelling adjunct to existing ART strategies, its routine clinical implementation requires validation through well-designed, large-scale prospective trials [44,46].

**Figure 3:**
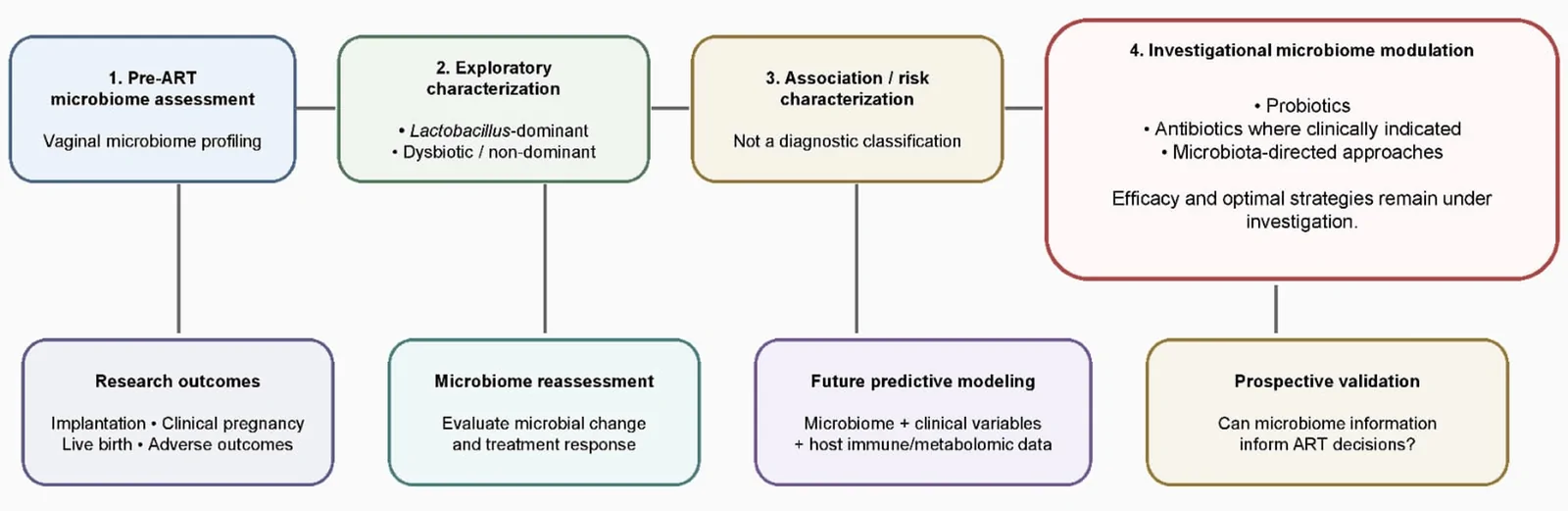
Proposed research framework for microbiome-informed fertility and ART decision-making ART: assisted reproductive technology Conceptual framework illustrating the potential integration of vaginal microbiome assessment into ART research and future clinical translation. The framework includes pre-ART microbiome profiling, exploratory characterization of microbial profiles, and investigation of associations with reproductive outcomes. Microbiome-directed interventions, including probiotics, antibiotics, and microbiota transplantation, are presented as investigational approaches rather than established therapeutic strategies. Integration of microbiome, clinical, artificial intelligence, and multi-omics data may facilitate the development of predictive models; however, their ability to guide treatment selection or embryo transfer decisions requires prospective validation. The framework is intended to represent potential research directions and should not be interpreted as a validated clinical algorithm. The figure has been created by Aishwarya Sreekumar Nair manually using the standard graphical tools available within BioRender (BioRender Inc., Toronto, Ontario, Canada).

A proposed framework for integrating microbiome profiling into ART workflows, including risk stratification, targeted intervention, and outcome prediction, is illustrated in Figure 3.

Limitations and Future Directions

Despite growing evidence linking the vaginal and endometrial microbiome with reproductive outcomes, important methodological and interpretive limitations remain. Much of the available evidence is observational, limiting causal inference and raising the possibility of reverse causation, whereby infertility, inflammation, hormonal treatment, or reproductive interventions may themselves alter the microbiome. Residual confounding by age, BMI, antibiotic exposure, sexual activity and semen exposure, contraceptive use, menstrual cycle timing, and hormonal treatment may also influence observed associations. Microbial composition can vary across the menstrual cycle and with reproductive exposures, emphasizing the need for standardized sampling and detailed recording of relevant clinical and behavioral factors.

Population heterogeneity is another important consideration. Microbiome associations may vary across ancestry, geography, diet, socioeconomic circumstances, healthcare practices, and environmental exposures and should not be interpreted as intrinsic race-based biological differences. Multicenter studies incorporating diverse populations are therefore needed to establish generalizability.

Substantial technical heterogeneity also limits comparison across studies. Differences in sample collection, DNA extraction, sequencing platforms, 16S rRNA target regions and primer selection, sequencing depth, taxonomic databases, and bioinformatic pipelines can alter microbial profiles and introduce batch effects. Low-biomass reproductive tract samples are particularly vulnerable to contamination, making appropriate negative controls and contamination-aware analytical approaches essential [48,49].

Interpretation is further complicated by the compositional nature of microbiome data, in which relative abundances do not necessarily reflect absolute microbial abundance. Moreover, there is no universally accepted definition or threshold for vaginal "dysbiosis", with studies using different combinations of *Lactobacillus *abundance, CSTs, diversity measures, and specific taxa. Standardized definitions and validated analytical approaches are therefore needed.

Finally, although observational studies suggest associations between microbiome profiles and ART outcomes, interventional evidence remains limited and inconsistent, particularly for live birth. A recent multicenter randomized trial found that treatment of abnormal vaginal microbiota with clindamycin and *L. crispatus *CTV-05 did not improve reproductive outcomes, including live birth [50]. Future research should prioritize adequately powered, prospective multicenter studies and randomized trials using standardized microbiome assessment and live birth as a primary patient-centered endpoint, before microbiome-guided interventions can be incorporated into routine reproductive care.

Vaginal-endometrial axis: is the uterus really sterile?

The traditional concept of a sterile uterine environment has been increasingly re-evaluated, prompting renewed interest in the potential existence and clinical relevance of an endometrial microbiome. While earlier assumptions were based largely on culture-based limitations, more recent molecular studies have detected microbial signatures within intrauterine compartments, raising important questions regarding their origin, stability, and functional significance in reproductive biology [51,52].

Despite these findings, the existence of a true resident uterine microbiome remains controversial. While some studies have identified bacterial signatures within intrauterine compartments, others have failed to detect consistent microbial presence in healthy pregnancies, particularly in mid-trimester amniotic fluid samples [53]. These conflicting observations have fueled an ongoing debate between the "in utero colonization" hypothesis and the traditional sterile womb model. Importantly, the absence of inflammatory responses in many of these studies suggests that even if microorganisms are present, they may exist in a controlled, non-pathogenic state rather than representing active infection [53].

A central mechanism proposed to explain the presence of microorganisms within the uterus is the microbial ascension hypothesis. Given the anatomical continuity between the vagina, cervix, and uterine cavity, it is plausible that microorganisms ascend from the lower genital tract into the endometrium. This process may be facilitated by sperm transport, uterine peristalsis, or disruption of cervical barrier function. Evidence supporting this hypothesis includes the detection of vaginal-associated taxa, such as *Lactobacillus*, *Gardnerella, *and *Prevotella, *within endometrial samples, suggesting a degree of microbial continuity along the reproductive tract [54]. Additionally, conditions such as endometriosis have been associated with the presence of non-native microbial species in the uterus, potentially reflecting translocation from distant mucosal sites, including the oral cavity [55].

However, the relationship between vaginal and endometrial microbiota is complex and not fully explained by simple microbial ascension. Several studies have demonstrated partial concordance between vaginal and uterine microbial communities, indicating that vaginal microbiota may influence, but not fully determine, the composition of the endometrial microbiome [54]. At the same time, other investigations have reported significant discordance, with the endometrium harboring distinct microbial signatures that differ from vaginal profiles. For example, endometrial samples have been shown to contain taxa such as *Propionibacterium *and *Corynebacterium*, which are not consistently dominant in vaginal communities [54]. These findings suggest that the uterus may represent a unique ecological niche shaped by local immune, hormonal, and metabolic factors rather than a simple extension of the vaginal microbiome.

The clinical relevance of the endometrial microbiome has been most prominently explored in the context of implantation and assisted reproduction. Studies have demonstrated that a *Lactobacillus*-dominant endometrial environment is associated with improved implantation rates, clinical pregnancy, and live birth outcomes, whereas dysbiotic profiles are linked to implantation failure [43]. Furthermore, pilot studies in women undergoing frozen embryo transfer have shown that distinct cervicovaginal microbial patterns are associated with differential implantation success, supporting the role of microbial composition as a potential biomarker for reproductive outcomes [56]. However, as discussed in the previous section, these associations remain largely observational, and causality has yet to be firmly established. Recent evidence has also highlighted potential age-related differences in endometrial microbial profiles among patients with recurrent reproductive failure, including variation in the presence and relative dominance of *Lactobacillus *species [57]. These findings further underscore the heterogeneity of the endometrial microbiome and the need for prospective studies to clarify its relationship with reproductive outcomes.

A major challenge in interpreting uterine microbiome data is the issue of sampling contamination, which is particularly relevant in low-biomass environments such as the endometrium. Contamination can arise from multiple sources, including the vaginal canal during transcervical sampling, laboratory reagents, and environmental DNA. Studies have demonstrated that commonly detected taxa, including *Lactobacillus*, may be present in negative controls, raising concerns about false-positive findings [58]. As a result, rigorous methodological controls, including the use of sterile sampling techniques and contamination-aware bioinformatics pipelines, are essential for the accurate characterization of the uterine microbiome. This limitation remains a major source of controversy and contributes to the ongoing debate regarding uterine sterility.

In this context, an emerging and clinically relevant concept is that of dual-compartment microbiome profiling, which integrates analysis of both vaginal and endometrial microbial communities. Given the partial concordance and potential discordance between these compartments, simultaneous assessment may provide a more comprehensive understanding of reproductive tract ecology. Dual-compartment profiling could identify discordant microbial patterns, such as a *Lactobacillus*-dominant vaginal microbiome with a dysbiotic endometrial profile, that may not be detected through single-site analysis. Such discrepancies may have important implications for implantation success and ART outcomes.

Furthermore, integration of vaginal and endometrial microbiome data with clinical and embryological parameters may enhance predictive modeling in reproductive medicine. By capturing both local and ascending microbial influences, dual-compartment approaches may improve risk stratification for implantation failure and guide personalized therapeutic interventions. Although still in its early stages, this approach represents a significant step toward precision reproductive medicine.

In conclusion, the concept of a sterile uterus is being progressively redefined in light of emerging microbiome research. While evidence suggests the presence of microbial signatures within the uterine environment, significant uncertainties remain regarding their origin, functional relevance, and clinical implications. The vaginal-endometrial axis represents a complex and dynamic system, shaped by microbial, immunological, and hormonal interactions. Future research focusing on standardized methodologies, longitudinal studies, and integrative multi-omics approaches will be essential to clarify the role of the uterine microbiome and to translate these findings into clinical practice.

Multi-omics and systems biology approaches

Advances in multi-omics technologies have transformed the study of the vaginal and endometrial microbiome, enabling a transition from descriptive taxonomic profiling toward integrative, systems-level understanding of host-microbe interactions. Traditional microbiological methods, while useful for detecting overt infections, are limited in their ability to characterize low-abundance or unculturable organisms and provide minimal insight into functional activity. In contrast, multi-omics approaches, including metagenomics, metatranscriptomics, metabolomics, and proteomics, offer complementary layers of information that collectively define the functional landscape of the reproductive tract microbiome [59].

Metagenomics, particularly through shotgun sequencing, provides comprehensive characterization of microbial communities by identifying the full repertoire of microbial DNA present within a sample. This approach enables high-resolution taxonomic classification and detection of rare or novel organisms. However, metagenomics alone cannot distinguish between metabolically active and inactive microbes. Metatranscriptomics addresses this limitation by analyzing RNA transcripts, thereby capturing real-time microbial gene expression and functional activity. Importantly, studies integrating metagenomic and metatranscriptomic data have demonstrated that microbial abundance does not necessarily correlate with transcriptional activity, highlighting the need for functional profiling in reproductive microbiome research [60]. Such discrepancies are particularly relevant in reproductive contexts, where low-abundance taxa may exert disproportionately large biological effects.

Metabolomics further extends this functional perspective by characterizing the biochemical products of microbial metabolism. Vaginal metabolomic profiling has identified distinct metabolic signatures associated with reproductive health and disease. *Lactobacillus*-dominant microbiota are characterized by high levels of lactic acid (including both D- and L-isomers), branched-chain amino acids, and other protective metabolites that maintain acidic pH and epithelial integrity. In contrast, dysbiotic communities produce elevated levels of SCFAs such as acetate, propionate, and butyrate, as well as biogenic amines including putrescine, cadaverine, and tyramine [61]. These metabolites are associated with increased inflammation, epithelial disruption, and impaired reproductive outcomes. The ratio of D- to L-lactic acid, in particular, has been implicated in modulating mucosal immunity and may influence susceptibility to infection and implantation failure.

Proteomic analyses provide an additional layer of insight by identifying host and microbial proteins involved in immune regulation and inflammatory responses. Studies of cervicovaginal fluid have identified a wide range of proteins, including cytokines, AMPs, and glycoproteins, that reflect the immunological state of the reproductive tract. Notably, alterations in protein glycosylation patterns have been associated with microbial composition and immune activity, suggesting a role for proteomic signatures as biomarkers of reproductive health [62]. These findings highlight the importance of integrating host-derived data with microbial profiles to fully understand the functional consequences of microbiome alterations.

A critical advancement in systems biology is the integration of multi-omics data with established reproductive biomarkers. For example, combining microbiome profiles with endometrial receptivity array (ERA) gene expression signatures may enhance the prediction of implantation windows and improve embryo transfer timing. Similarly, hormonal profiling, particularly levels of estrogen and progesterone, plays a key role in shaping microbial composition and function, influencing glycogen availability, epithelial turnover, and immune responses. Genetic polymorphisms in host immune pathways, including Toll-like receptor (TLR) signaling and cytokine genes, may further modulate host-microbiome interactions, contributing to interindividual variability in reproductive outcomes. Integrating these factors into multi-omic frameworks allows for a more comprehensive understanding of reproductive physiology and pathology.

The convergence of multi-omics data has paved the way for the development of AI-driven predictive models in reproductive medicine. Machine learning algorithms, including support vector machines, random forests, and neural networks, have been applied to integrate microbiome, metabolomic, and immunological data to predict ART outcomes. In a recent study, a supervised machine learning model combining vaginal microbiome composition with host inflammatory markers demonstrated high accuracy in predicting IVF success, with key microbial taxa and cytokines identified as major contributors to model performance [63]. Such approaches enable the identification of complex, non-linear relationships that are not apparent through traditional statistical methods.

Importantly, these developments signal a paradigm shift from association-based studies toward predictive reproductive modeling. While earlier research primarily focused on identifying correlations between microbial composition and reproductive outcomes, multi-omics integration allows for the construction of predictive frameworks that can inform clinical decision-making. For instance, predictive models may identify patients at high risk of implantation failure based on combined microbial, metabolic, and immunological signatures, enabling targeted interventions prior to ART cycles.

Despite these advances, several challenges remain. Multi-omics studies are resource-intensive, require standardized methodologies, and generate high-dimensional data that demand sophisticated analytical approaches. Additionally, variability in sample collection, sequencing platforms, and data processing pipelines can limit reproducibility. Nevertheless, ongoing improvements in computational methods and the increasing availability of large-scale datasets are expected to address these limitations.

In conclusion, multi-omics and systems biology approaches are redefining the landscape of reproductive microbiome research. By integrating multiple layers of biological information, these technologies provide a comprehensive framework for understanding the complex interplay between microbial communities and host physiology. The transition from descriptive to predictive modeling represents a critical step toward precision reproductive medicine, with the potential to improve fertility outcomes through individualized diagnostic and therapeutic strategies.

Therapeutic modulation: from probiotics to microbiome transplantation

Therapeutic modulation of the vaginal microbiome has emerged as a promising strategy for improving reproductive outcomes, particularly in the context of infertility and ART. As discussed previously, dysbiosis is associated with impaired implantation and adverse reproductive outcomes, prompting interest in targeted interventions aimed at restoring a *Lactobacillus*-dominant reproductive tract environment [43,44].

Antibiotics in ART: Benefit Versus Harm

The use of antibiotics in ART cycles remains controversial. While antibiotic therapy may be beneficial in specific conditions such as CE or confirmed pathogenic infections, its routine empirical use is not supported by robust evidence. A Cochrane review demonstrated no clear improvement in clinical pregnancy or live birth rates with prophylactic antibiotics at the time of embryo transfer [64]. Furthermore, antibiotic exposure may disrupt commensal microbial communities and reduce *Lactobacillus *dominance, potentially counteracting reproductive benefits [65]. Therefore, antibiotic therapy should be individualized and reserved for clearly defined infectious indications.

Probiotics: Oral Versus Vaginal Administration

Probiotic supplementation is one of the most extensively studied microbiome-directed therapies. Specific strains such as *Lactobacillus rhamnosus *GR-1 and *Lactobacillus reuteri *RC-14 have demonstrated efficacy in restoring vaginal microbial balance and reducing recurrence of BV [66,67]. Evidence suggests that both oral and vaginal routes can modulate the vaginal microbiome, with comparable effectiveness in many studies [66]. Emerging data further indicate that probiotic-induced restoration of *Lactobacillus *dominance may improve local immune regulation and epithelial integrity, thereby indirectly supporting implantation [67].

Vaginal Microbiota Transplantation

Vaginal microbiota transplantation is an emerging therapeutic strategy for refractory dysbiosis. A landmark pilot study demonstrated that vaginal microbiota transplantation from healthy donors resulted in the long-term remission of recurrent BV and the restoration of *Lactobacillus*-dominant microbiota [68]. Although evidence in infertility and ART populations remains limited, the mechanistic rationale is strong, particularly in cases of persistent dysbiosis unresponsive to conventional therapies.

Prebiotics and Synbiotics

Prebiotics and synbiotics represent adjunctive strategies aimed at promoting the growth and stability of beneficial microbial communities. Prebiotics such as glycogen-derived substrates and oligosaccharides selectively enhance *Lactobacillus *proliferation, while synbiotics combine these substrates with probiotic strains to achieve synergistic effects [69]. These approaches may improve microbial resilience and reduce the recurrence of dysbiosis, although high-quality fertility-specific data remain limited.

Hormonal Modulation of the Microbiome

Hormonal regulation plays a central role in shaping the vaginal microbiome. Estrogen promotes glycogen deposition in vaginal epithelial cells, which serves as a substrate for *Lactobacillus *metabolism. Clinical studies have demonstrated that estrogen-rich states are associated with *Lactobacillus*-dominant microbiota, whereas hypoestrogenic states are linked to increased microbial diversity and dysbiosis [70,71]. In ART cycles, controlled ovarian stimulation and hormonal supplementation may therefore indirectly influence microbial composition.

Lifestyle Factors as Modifiable Interventions

Lifestyle factors, including diet, stress, and hygiene practices, influence the vaginal microbiome and represent modifiable therapeutic targets. Dietary patterns rich in micronutrients and fiber have been associated with improved reproductive outcomes [72]. Chronic stress, mediated through endocrine pathways, can alter microbial homeostasis [73]. Additionally, certain hygiene practices have been linked to the disruption of vaginal microbial balance and increased risk of dysbiosis [67].

Precision Microbiome Correction Before Embryo Transfer

An emerging concept in reproductive medicine is precision microbiome correction prior to embryo transfer. This involves pre-ART microbiome screening followed by targeted intervention in cases of dysbiosis. Observational evidence indicates that restoration of *Lactobacillus*-dominant profiles is associated with improved implantation and pregnancy outcomes, supporting this strategy [43,44]. Frozen embryo transfer cycles offer a particularly valuable window for such interventions.

Ongoing Clinical Trials and Future Directions

Recent clinical trials have begun to explore microbiome-directed therapies in reproductive medicine. Studies evaluating probiotics, combined microbiome and vitamin D modulation, and targeted antimicrobial therapy have demonstrated promising but heterogeneous results [67,74]. Additionally, emerging trials are investigating microbiome transplantation and multi-omic-guided interventions as novel therapeutic approaches.

Toward Microbiome-Directed Fertility Optimization

Collectively, these findings support a paradigm shift toward microbiome-directed fertility optimization. Integration of microbiome screening, targeted correction, and individualized therapeutic strategies may enhance reproductive outcomes and represents a key step toward precision reproductive medicine. Current microbiome-directed therapeutic strategies and their clinical implications are summarized in Table 2.

**Table 2:** Microbiome-directed therapeutic strategies in reproductive medicine: mechanisms, evidence, and clinical applications ART: assisted reproductive technology; BV: bacterial vaginosis; VMT: vaginal microbiota transplantation; RCTs: randomized controlled trials

Intervention	Mechanism of action	Evidence level	Clinical application in ART	Limitations	Reference(s)
Antibiotics	Eradication of pathogenic bacteria	Moderate (condition-specific)	Chronic endometritis, infection-related infertility	Disruption of beneficial microbiota, resistance risk	[64,65]
Probiotics (oral/vaginal)	Restoration of *Lactobacillus *dominance	Moderate to high	BV treatment, pre-ART microbiome optimization	Strain-specific variability, inconsistent colonization	[66,67]
VMT	Reconstitution of healthy microbiome	Emerging	Refractory dysbiosis	Limited ART-specific data, regulatory concerns	[68]
Prebiotics/synbiotics	Promote growth of beneficial bacteria	Emerging	Microbiome stabilization	Limited clinical trials in fertility settings	[69]
Hormonal modulation	Estrogen-driven glycogen production supports *Lactobacillus*	Moderate	ART cycles, hormonal therapy	Indirect effects, variability among patients	[70,71]
Lifestyle interventions	Modulation via diet, stress, hygiene	Low to moderate	Adjunct fertility optimization	Non-specific effects, limited direct evidence	[72,73]
Precision microbiome correction	Targeted therapy prior to embryo transfer	Emerging	Personalized ART protocols	Lack of standardized protocols, limited RCTs	[43,44]
Combined therapies (e.g., probiotics + vitamin D)	Synergistic microbiome and immune modulation	Emerging	Recurrent implantation failure	Heterogeneous evidence	[74]

Clinical translation, gaps, and future directions

Despite growing recognition of the reproductive microbiome as a potential determinant of fertility outcomes, its translation into routine clinical practice remains limited. This gap reflects ongoing challenges related to methodological variability, lack of standardized diagnostic criteria, and insufficient interventional evidence [75]. Addressing these barriers is essential to move from observational associations toward clinically actionable applications.

Sampling-related challenges are particularly pronounced in the study of the endometrial microbiome. Given its low microbial biomass, endometrial sampling is highly susceptible to contamination from the vaginal canal, laboratory reagents, and environmental sources. Transcervical sampling techniques, although practical, inherently risk cross-contamination, potentially confounding microbial signatures attributed to the uterine environment [76]. These limitations underscore the need for standardized sampling protocols, contamination controls, and validated methodologies before widespread clinical implementation.

Another critical gap is the lack of large-scale, longitudinal randomized controlled trials. While numerous observational studies have demonstrated associations between microbiome profiles and reproductive outcomes, causal relationships remain inadequately established. Current evidence is largely derived from small cohorts with heterogeneous populations and methodologies, limiting generalizability [77]. Well-designed randomized controlled trials are essential to determine whether microbiome-targeted interventions can meaningfully improve fertility outcomes and to define optimal therapeutic strategies.

Regulatory considerations further complicate clinical translation. Microbiome-based diagnostics and therapeutics, including probiotics and vaginal microbiota transplantation, fall within evolving regulatory frameworks that vary across jurisdictions. Issues related to safety, donor screening, quality control, and long-term outcomes must be addressed before such interventions can be widely adopted. In parallel, ethical concerns, including informed consent, data privacy, and the potential misuse of microbiome data, require careful consideration. Ethical frameworks specific to microbiome research emphasize transparency, equitable access, and responsible use of biological data [78].

Cost-effectiveness represents another important barrier, particularly in ART settings. Advanced sequencing technologies and multi-omics analyses remain expensive and are not routinely available in most fertility clinics. Additionally, the turnaround time for microbiome analysis may not align with the time-sensitive nature of ART cycles, limiting real-time clinical utility [79]. Economic evaluations are therefore necessary to determine whether microbiome-guided interventions provide sufficient benefit to justify their cost.

In this context, a microbiome-based reproductive risk stratification model may offer a pragmatic pathway toward clinical integration. Such a model would incorporate microbial composition (e.g., CST classification), functional signatures (metabolomic and proteomic profiles), and host factors (immune and hormonal parameters) to categorize patients into low-, intermediate-, and high-risk groups for implantation failure or adverse reproductive outcomes. Preliminary studies have demonstrated that microbiome profiling prior to ART can stratify patients based on probability of pregnancy, highlighting its potential as a predictive tool [80].

Building on this framework, a roadmap for integrating microbiome profiling into fertility clinics can be proposed. Initial steps include standardized microbiome screening prior to ART cycles, followed by targeted interventions in cases of dysbiosis. Integration with existing diagnostic tools, such as endometrial receptivity assays and hormonal profiling, may further enhance predictive accuracy. Over time, incorporation of AI-driven models could enable real-time decision support, guiding embryo transfer timing and personalized therapeutic strategies.

Thus, while the reproductive microbiome holds significant promise as a diagnostic and therapeutic target, its clinical translation requires overcoming substantial methodological, regulatory, and economic challenges. Future research should prioritize standardization, large-scale interventional studies, and integration of multi-omics data to enable the development of robust, clinically actionable models for fertility optimization.

## Conclusions

The vaginal microbiome represents an important and evolving dimension of reproductive health, with growing evidence linking microbial composition and function to natural conception and ART outcomes. *Lactobacillus*-dominant profiles are generally associated with favorable reproductive outcomes, whereas dysbiosis may contribute to impaired sperm function, altered immune regulation, reduced endometrial receptivity, implantation failure, and pregnancy loss. Advances in multi-omics and AI may further enable the development of microbiome-based biomarkers and personalized reproductive risk assessment.

Despite this promise, translation into routine clinical practice remains limited by methodological heterogeneity, inconsistent definitions of dysbiosis, sampling-related challenges, and insufficient interventional evidence. Future research should prioritize standardized methodologies and well-designed prospective and randomized studies to establish causality and determine whether targeted microbiome modulation improves clinically meaningful outcomes. Careful validation will be essential before microbiome profiling and therapeutic interventions can be integrated into routine fertility care.

## Appendices

**Table 3:** Database-specific search strategies Search date: March 5, 2026 Publication period: January 2015 to February 2026 Reference management and duplicate removal: Zotero Version 10 (Corporation for Digital Scholarship, Vienna, Virginia, United States)

Database	Reconstructed search strategy
PubMed/MEDLINE	("vaginal microbiome"[Title/Abstract] OR "vaginal microbiota"[Title/Abstract] OR "community state type"[Title/Abstract] OR dysbiosis[Title/Abstract] OR Lactobacillus[Title/Abstract]) AND (fertility[Title/Abstract] OR "natural conception"[Title/Abstract] OR "in vitro fertilization"[Title/Abstract] OR IVF[Title/Abstract] OR ICSI[Title/Abstract] OR "intracytoplasmic sperm injection"[Title/Abstract] OR "assisted reproductive technolog"[Title/Abstract] OR "endometrial microbiome"[Title/Abstract] OR implantation[Title/Abstract] OR "implantation failure"[Title/Abstract] OR "pregnancy outcome*"[Title/Abstract] OR "reproductive outcome*"[Title/Abstract])**
Scopus	TITLE-ABS-KEY(("vaginal microbiome" OR "vaginal microbiota" OR "community state type" OR dysbiosis OR Lactobacillus) AND (fertility OR "natural conception" OR "in vitro fertilization" OR IVF OR ICSI OR "intracytoplasmic sperm injection" OR "assisted reproductive technolog" OR "endometrial microbiome" OR implantation OR "implantation failure" OR "pregnancy outcome*" OR "reproductive outcome*"))**
Web of Science Core Collection	TS=(("vaginal microbiome" OR "vaginal microbiota" OR "community state type" OR dysbiosis OR Lactobacillus) AND (fertility OR "natural conception" OR "in vitro fertilization" OR IVF OR ICSI OR "intracytoplasmic sperm injection" OR "assisted reproductive technolog" OR "endometrial microbiome" OR implantation OR "implantation failure" OR "pregnancy outcome*" OR "reproductive outcome*"))**
Cochrane Library	("vaginal microbiome" OR "vaginal microbiota" OR "community state types" OR dysbiosis OR Lactobacillus) AND (fertility OR "natural conception" OR "in vitro fertilization" OR IVF OR ICSI OR "assisted reproductive technology" OR "endometrial microbiome" OR implantation OR "implantation failure" OR "pregnancy outcomes" OR "reproductive outcomes")
